# Study on the serum level of CoQ10B in patients with Moyamoya disease and its mechanism of affecting disease progression

**DOI:** 10.1590/0004-282X-ANP-2021-0002

**Published:** 2022-05-20

**Authors:** Jian MA, Xudong FU, Shaolong ZHOU, Enping MENG, Zhuo YANG, Hengwei ZHANG

**Affiliations:** 1 Zhengzhou University, The Fifth Affiliated Hospital of Zhengzhou, Department of Neurosurgery, Henan, China.

**Keywords:** Moyamoya Disease, Endothelial Progenitor Cells, Mitochondria, Doença de Moyamoya, Células Progenitoras Endoteliais, Mitocôndrias

## Abstract

Background: At present, the etiology and pathogenesis of Moyamoya disease (MMD) are not completely clear. Patients are usually diagnosed after cerebrovascular events. Therefore, it is of great clinical significance to explore the predictive factors of MMD. Objective: This study aimed to investigate the serum level of CoQ10B, the amount of endothelial progenitor cells (EPCs), and mitochondrial function of EPCs in MMD patients. Methods: Forty-one MMD patients and 20 healthy controls were recruited in this study. Patients with MMD were divided into two groups: Ischemic type (n=23) and hemorrhagic type (n=18). Blood samples were collected from the antecubital vein and analyzed by CoQ10B ELISA and flow cytometry. Measures of mitochondrial function of EPCs include oxygen consumption rate (OCR), mitochondrial membrane potential, Ca^2+^ concentration, adenosine triphosphatases activity and ROS level. Results: The serum CoQ10B level in MMD patients was significantly lower than that in healthy controls (p<0.001). The relative number of EPCs in MMD patients was significantly higher than that in healthy controls (p<0.001). Moreover, the OCR, mitochondrial membrane potential and ATPase activity were decreased and the Ca^2+^ and reactive oxygen species levels were increased in MMD patients (p<0.001). Conclusions: Our results showed obviously decreased serum CoQ10B level and increased EPCs number in patients with MMD compared with healthy patients, and the mitochondria function of EPCs in MMD patients was abnormal.

## RESUMO

Antecedentes: No momento, a etiologia e a patogênese da doença de Moyamoya (DMM) não são completamente claras. Os pacientes geralmente são diagnosticados após eventos cerebrovasculares. Sendo assim, é de grande importância clínica explorar os fatores preditivos de DMM. Objetivo: Este estudo teve como objetivo investigar o nível sérico de CoQ10B, a quantidade de células progenitoras endoteliais (CPE) e a função mitocondrial de CPE em pacientes com DMM. Métodos: Quarenta e um pacientes com DMM e 20 controles saudáveis foram recrutados neste estudo. Aqueles com DMM foram divididos em dois grupos: tipo isquêmico (n=23) e tipo hemorrágico (n=18). Amostras de sangue foram coletadas da veia antecubital e analisadas por CoQ10B Ensaio de Imunoadsorção Enzimática (ELISA) e citometria de fluxo. As medidas da função mitocondrial de CPE incluem taxa de consumo de oxigênio (TCO), potencial de membrana mitocondrial, concentração de Ca^2+^, atividade de adenosina trifosfatases (ATPase) e nível de espécies reativas de oxigênio (ROS). Resultados: O nível sérico de CoQ10B em pacientes com DMM foi significativamente menor do que em controles saudáveis (p<0,001). O número relativo de CPE em pacientes com MMD foi significativamente maior do que em controles saudáveis (p<0,001). Além disso, a TCO, o potencial de membrana mitocondrial e a atividade ATPase diminuíram e os níveis de Ca^2+^e ROS aumentaram em pacientes com MMD (p<0,001). Conclusões: Nossos resultados mostraram obviamente diminuição do nível sérico de CoQ10B e aumento do número de CPE em pacientes com DMM em comparação com pacientes saudáveis, e a função mitocondrial de CPE em pacientes com DMM estava anormal.

## INTRODUCTION

Moyamoya disease (MMD) is a chronic progressive cerebrovascular disease, which can be divided into three types: ischemic, hemorrhagic, and asymptomatic^1^ ^,^ ^2^ ^,^ ^3^. It is usually caused by stenosis or occlusion at the end of internal carotid artery caused by various reasons. Although the incidence rate of MMD is high in cerebral hemorrhage and cerebral ischemia, the mechanism of its occurrence and development is not clear.

Endothelial progenitor cells (EPCs) are the main cell components in angiogenesis and are considered to be an important participant in the occurrence and development of MMD^4^ ^,^ ^5^. As precursor cells, EPCs can directly differentiate into vascular endothelial cells, which are not only involved in the formation of early embryonic blood vessels, but also the most closely related stem cells in adults. Previous studies have found that MMD patients have a higher EPCs number than the healthy controls^6^ ^,^ ^7^ ^,^ ^8^. Other studies also show that the number of EPCs in MMD patients is lower than that in healthy controls^9^ ^,^ ^10^. Besides, Bao et al.^11^ have reported that MMD patients have higher EPCs counts than the healthy controls, but it did not reach statistical significance. Further experiments are needed to confirm the changes of EPCs in MMD patients.

At present, the recognized EPCs surface markers are CD34, CD133, and VEGFR-2^12^. Thus, we defined EPCs as CD34^+^CD133^+^VEGFR-2^+^ cells in this study. Coenzyme Q10 (ubiquinone or coenzyme Q10) is a lipid soluble component that exists in the cell membranes and acts as a carrier for moving electrons and protons^13^. A new study reported that intravenous administration of CoQ10 has a persistent neuroprotective potential in rat stroke model^14^. In addition, CoQ10 can decrease histological organ damage in sepsis^15^ ^,^ ^16^, be used as a potential treatment for diseases related to the mitochondrial respiratory chain due to its antioxidant effect^17^, and attenuate high glucose-induced EPCs apoptosis^18^. CoQ10B (coenzyme Q10, homolog B) is an anti-apoptosis enzyme stored in the mitochondria of cells and belongs to the CoQ10 family. CoQ10B is an important biological cofactor, which can increase the concentration of mitochondria in brain and play a neuroprotective role^19^. Moreover, Peng et al.^20^ have reported a significantly lower serum CoQ10B level in MMD patients than that in healthy controls.

Several studies have reported the abnormal number of EPCs in MMD patients, but only one reported the abnormal expression of CoQ10B in MMD patients. Thus, this study verified serum CoQ10B level, number of EPCs, and mitochondrial function of EPCs in MMD patients to provide the basis for molecular targeted therapy of MMD.

## METHODS

### Patient selection

The study was approved by medical ethics committee of The Fifth Affiliated Hospital of Zhengzhou University. All the participants were informed of the study and signed an informed consent. Forty-one adult MMD patients admitted to The Fifth Affiliated Hospital of Zhengzhou University from June 2018 to March 2020 were selected as the case group, all of which were confirmed by three-dimensional digital subtraction angiography. The results of cerebral angiography were regarded as the “gold standard” for the diagnosis of MMD. The specific criteria were: I, stenosis or occlusion at the end of the internal carotid artery and/or at the beginning of anterior cerebral artery and middle cerebral artery; II, abnormal vascular network in skull base; III, bilateral lesions.

The exclusion criteria for MMD patients were: I, certain known diseases, such as autoimmune diseases, meningitis, brain tumors, type I neurofibroma, Down’s syndrome and brain radiotherapy, which may also cause similar “Moyamoya vessels” in some cerebral vessels during the pathological process, called “quasi Moyamoya disease” or “Moyamoya syndrome”; II, severe atherosclerosis, hypertension, cardiopulmonary and renal insufficiency, contrast agent allergy, and other contraindications of cerebral angiography; III, patients and their families disagree with cerebral angiography. The 41 MMD patients included 23 cases of ischemic type and 18 cases of hemorrhagic type.

Twenty healthy people who came to our hospital for physical examination in the same period were selected as the healthy control group. They were mainly matched based on age and sex and had normal blood counts, biochemical values, electrocardiogram, chest X-ray, color Doppler abdominal ultrasound and color Doppler transcranial ultrasound.

### Peripheral blood sample collection

Peripheral blood samples of MMD patients before treatment and of the healthy control group were collected with Ficoll-Paque PLUS (Amersham Corporation). Mononuclear cells were isolated using density-gradient centrifugation.

### Detection of Coenzyme Q10 Homolog B by Enzyme-Linked Immunosorbent Assay

Serum CoQ10B level was detected according to the instructions of the ELISA kit (Cusabio, USA). The optical density was read on a plate reader with wavelength of 450 nm. The concentration of CoQ10B was calculated based on the standard curve.

### Detection of endothelial progenitor cells by flow cytometry

Collected mononuclear cells were diluted in PBS buffer (10^7^ cells/mL). Fc receptor antibody was added for blocking, then PE-CD133 antibody, FITC-CD34 antibody and APC-VEGFR-2 antibody were added. The antibody-labeled cells were washed and detected by flow cytometry. Cells positive for the three antibody were considered EPCs. The relative number of EPCs in mononuclear cells was recorded.

### Measurements of oxygen consumption rate

Seahorse XF24 Extracellular Flux Analyzer was used to detect OCRs. First, EPCs were seeded onto a XF 24-well cell culture microplate (2×10^4^). After obtaining the basic OCR, oligomycin (sigma Aldrich), 2,4-dinitrophenol (sigma Aldrich), rotenone (sigma Aldrich), and antimycin (sigma Aldrich) were added to each well to measure OCRs.

### Detection of mitochondrial membrane potential

In depolarized cells, labeling of the mitochondria with tetramethylrhodaminemethyl ester (TMRM) disappears, and the red fluorescence can be used as an indicator of MMP. Phenol red-free medium containing 500 nM TMRM was used to replace the medium. The plates were incubated for 1 hour at 37°C and washed with PBS for 3 times. Fluorescence signals were detected using a microscope (Olympus), and more than 100 cells in each group were analyzed.

### Intracellular Ca^2+^concentrations

EPCs were cultured in 6-well plates for 12 hours, then the calcium fluorescent probe was added to the cells and the reaction time was 30 minutes at room temperature. Calcium concentration changes in the EPCs were detected by confocal microscopy.

### Reactive oxygen species level

DCFH-DA was used to detect the level of ROS. In short, about 10^6^ cells were incubated with DCFH-DA in the dark at 37°C for 30 min. Flow cytometry and Cell Quest software (Becton Dickinson) were used to detect and analyze the fluorescence.

### Adenosine triphosphatases activity

According to the manufacturer’s instructions, the ATPase activity was detected by an ATP Colorimetric/Fluorometric Assay Kit (Cat# MAK190-1, Sigma).

### Statistical analysis

All the clinical data are presented as mean±standard deviation (SD), and statistical analysis was carried out with SPSS 25.0. Chi-square (χ2) test was used for categorical variables and independent Student’s t tests was used for continuous variables. P value less than 0.05 was considered to be statistically significant.

## RESULTS

### Basic clinical information

There were 12 males and 8 females in the control group, aged between 18 to 62 years, with an average age of (47.35±11.16) years. In the MMD patients group, there were 26 males and 15 females with an average age of (48.54± 6.66) years (range of 29-61 years). There was no significant difference in gender (χ^2^=0.067, p=0.796) and age (t=-0.439, p=0.664) between groups. In the ischemic type there were 15 males and 8 females aged 32 to 61 years (average 48.04±5.87 years), with diabetes mellitus in 4 cases, smoking history in 12 cases, and conservative treatment in 10 cases and surgical treatment in 13 cases. In the hemorrhagic type there were 11 males and 7 females with an average age of 49.17±7.69 years and range of 29-60 years. There were 3 cases of diabetes mellitus and 7 cases of smoking history. The treatment methods were conservative treatment in 7 cases and surgical treatment in 11 cases. There were no significant differences in gender (χ^2^=0.073, p=0.786), age (t=18.533, p=0.615), incidence of diabetes (χ^2^=0.004, p=0.951), smoking history (χ^2^=0.717, p=0.397), and treatment methods (χ^2^=0.088, p=0.767) between ischemic and hemorrhagic patients.

### Serum Coenzyme Q10 Homolog B levels

**Figure 1.**
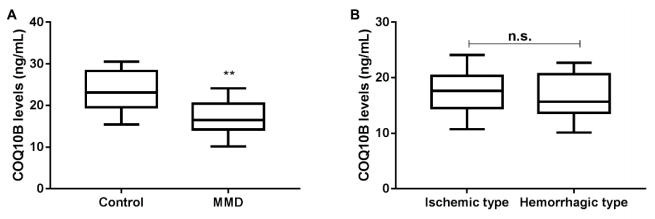
Serum levels of CoQ10B in control and Moyamoya disease patients. (A) Comparison of serum levels of CoQ10 between control group and Moyamoya disease patients group. (B) Comparison of serum levels of CoQ10B between Moyamoya disease patients with ischemic type and hemorrhagic type.

Serum CoQ10B level of the MMD group (17.07±3.61 ng/mL) was lower than that of the control group (23.60±4.46 ng/mL) on the day of admission (p<0.001, Figure 1A). There was no significant difference in serum CoQ10B levels between the ischemic and hemorrhagic groups (17.40±3.57 ng/mL) vs (16.64±3.72 ng/mL), (p>0.05, Figure 1B).

**p<0.001 vs Control. MMD: Moyamoya disease.

### Endothelial progenitor cells in peripheral blood

**Figure 2.**
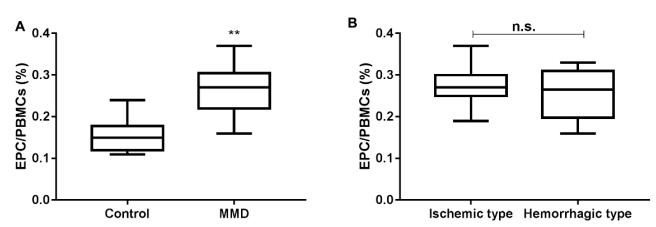
Relative amounts of endothelial progenitor cellsin control and Moyamoya disease patients. (A) Comparison of the relative number of endothelial progenitor cellsin peripheral blood between the control group and the Moyamoya disease patients group. (B) Comparison of the relative number of endothelial progenitor cells in peripheral blood between the MMD patients with ischemic type and hemorrhagic type.

The relative number of EPCs in peripheral blood of the MMD group (0.26±0.05% of PBMCs) on the day of admission was significantly higher than that of the control group (0.15±0.04% of PBMCs) (p<0.001, Figure 2A). There was also no significant difference in the relative number of EPCs in peripheral blood between the ischemic and hemorrhagic groups (p>0.05, Figure 2B).

**p<0.001 vs Control. MMD: Moyamoya disease; EPCs: endothelial progenitor cells.

### Correlation of mitochondrial function of endothelial progenitor cells from Moyamoya disease patients

**Figure 3.**
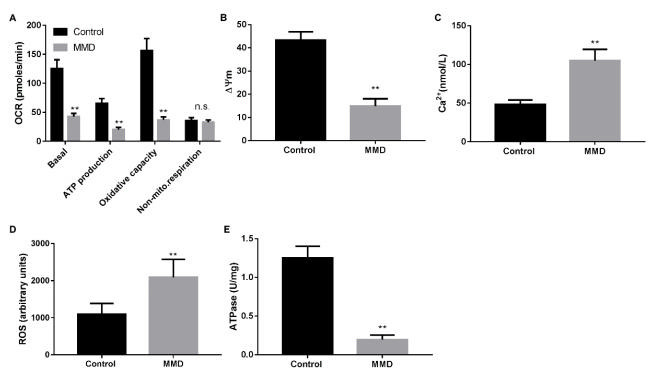
Mitochondrial function of endothelial progenitor cells from control and Moyamoya disease patients. (A) Oxygen consumption rate. (B) Mitochondrial membrane potential. (C) Ca^2+^ concentration. (D) reactive oxygen species level. (E) adenosine triphosphatases activity.

The mitochondrial function change of EPCs in MMD patients was evaluated through OCR. Compared with healthy controls, EPCs from MMD patients had decreased basal respiratory rate, ATP level, and oxidative capacity (Figure 3A), while the antimycin-insensitive OCR was mildly but non-significantly decreased in EPCs of the MMD. Moreover, the mitochondrial membrane potential (ΔΨm) and ATPase activity were decreased, and the Ca^2+^ and ROS levels were increased in MMD EPCs (Figures 3B to 3E, p<0.001), which indicated abnormalities in mitochondrial function of MMD EPCs.

**p<0.001 vs Control. MMD: Moyamoya disease; EPCs: endothelial progenitor cells.

## DISCUSSION

Studies have shown that the development of MMD is accompanied by angiogenesis. The pathogenesis of MMD is related to EPC abnormality^6^. Currently, it is generally believed that the characteristic surface antigens of EPCs are CD133, CD34 and VEGFR-2. Therefore, we defined EPCs as CD34^+^CD133^+^VEGFR-2^+^ cells. Our study found a significantly higher number of EPCs in MMD group than in control group, which was consistent with previous studies^6^ ^,^ ^7^. Previous studies have found that the number of EPCs in patients after superficial temporal artery-middle cerebral artery (STA-MCA) anastomosis was significantly lower than before operation, and the postoperative cerebral angiography was also reduced^21^, which indicated that the increased number of EPCs in patients is one of the pathogenic mechanisms of MMD.

Through a literature search we found that results on the level of EPCs in MMD patients are controversial. Kim et al.^9^ reported decreased levels and defective function of EPCs in MMD patients. While MMD patients recruited in Kim et al.’s study were children, the patients recruited in our study were adults. Jung et al.^10^ reported that EPC functional activity was impaired in patients with MMD. The authors found that the colony-forming units (CFU) of EPCs isolated from MMD patients was obviously decreased compared with that of healthy controls, which was different from the relative quantity of EPCs in peripheral blood detected by flow cytometry in our study. This further proves the reliability of our results.

Mitochondria are the powerhouse of cells. They are involved in the production of ATP, regulation of intracellular Ca^2+^, the production and elimination of ROS, regulation of apoptotic cell death, and activation of the protease caspase family, which play an important role in cell proliferation, differentiation, survival, and homeostasis^22^. A large number of studies have reported that CoQ10 deficiency leads to cancer^23^ and other diseases^24^, and that CoQ10 supplementation can significantly alleviate disease progression^25^ ^,^ ^26^ ^,^ ^27^. CoQ10B belongs to the CoQ10 family and exists in mitochondria of most eukaryotes, which are an important part of the electron transport chain. Here, we found that the serum CoQ10B level was significantly lower in MMD patients than in controls, which indicated that CoQ10B deficiency may be one of the pathogenesis of MMD.

CoQ10 supplements can play a therapeutic role in many diseases by improving mitochondrial dysfunction^28^ ^,^ ^29^ ^,^ ^30^ ^,^ ^31^. Choi et al.^32^ found that the morphology and function of mitochondria in ECFCs of MMD patients were abnormal, suggesting that MMD may be a mitochondrial-related disease. In addition, our study found abnormalities in mitochondrial function of MMD EPCs, which further suggested that CoQ10B may be involved in the MMD disease by affecting mitochondrial function. As we all know, mitochondrial dysfunction leads to apoptosis. Whether the reduced CoQ10B level in MMD patients affect EPCs proliferation and thus decrease the number of EPCs needs further experimental verification.

Our study found that the mitochondria of EPCs from MMD patients exhibited functional abnormalities, the serum CoQ10B level was significantly lower, and the relative number of EPCs was significantly higher in MMD patients than in controls, which provide the basis for the molecular targeted therapy of MMD. However, there was no significant difference in CoQ10B level and number of EPCs between ischemic and hemorrhagic type MMD patients. Maybe the number of cases is too small or there is no significant difference between the two groups, therefore further studies with larger samples are needed. Moreover, the correlation between CoQ10B level and mitochondrial dysfunction in MMD patients should be verified.

In conclusion, serum CoQ10B level was obviously decreased and EPCs number was increased in patients with MMD compared to controls, and the mitochondria function of EPCs in MMD patients was abnormal.
